# Nanophotonic coherent light–matter interfaces based on rare-earth-doped crystals

**DOI:** 10.1038/ncomms9206

**Published:** 2015-09-14

**Authors:** Tian Zhong, Jonathan M. Kindem, Evan Miyazono, Andrei Faraon

**Affiliations:** 1 T. J. Watson Laboratory of Applied Physics, California Institute of Technology, 1200 East California Boulevard, MC 107-81, Pasadena, California 91125, USA

## Abstract

Quantum light–matter interfaces connecting stationary qubits to photons will enable optical networks for quantum communications, precise global time keeping, photon switching and studies of fundamental physics. Rare-earth-ion-doped crystals are state-of-the-art materials for optical quantum memories and quantum transducers between optical photons, microwave photons and spin waves. Here we demonstrate coupling of an ensemble of neodymium rare-earth-ions to photonic nanocavities fabricated in the yttrium orthosilicate host crystal. Cavity quantum electrodynamics effects including Purcell enhancement (*F*=42) and dipole-induced transparency are observed on the highly coherent ^4^I_9/2_–^4^F_3/2_ optical transition. Fluctuations in the cavity transmission due to statistical fine structure of the atomic density are measured, indicating operation at the quantum level. Coherent optical control of cavity-coupled rare-earth ions is performed via photon echoes. Long optical coherence times (*T*_2_∼100 μ*s*) and small inhomogeneous broadening are measured for the cavity-coupled rare-earth ions, thus demonstrating their potential for on-chip scalable quantum light–matter interfaces.

## Abstract (web summary)

Quantum light–matter interfaces connecting stationary qubits to photons are fundamental elements of future quantum optical networks. Here, the authors report a quantum interface based on highly coherent rare-earth ions—the solid-state qubits—coupled to a nanophotonic cavity fabricated in the host crystal.

## Graphical abstract

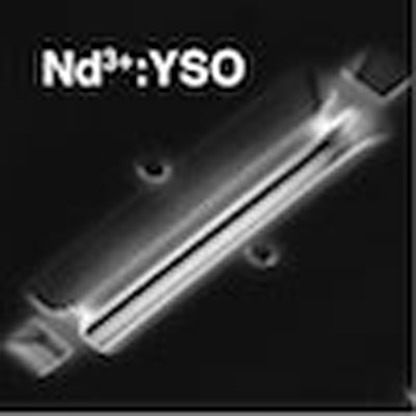

Quantum light–matter interfaces (QLMIs) are quantum devices composed of light emitters with quantum states that can be controlled via optical fields and entangled to photons^1^,^2^. They enable distribution of quantum entanglement over long distances in optical quantum networks for quantum communications^1^. Quantum networks of atomic clocks have also been proposed for precise global time keeping and studies of fundamental physics^2^. Realizing QLMIs requires control of light and matter at the single atom and single photon level, which enables optoelectronic devices such as optical modulators and nonlinear optical devices operating at the most fundamental level^3^. QLMIs are also expected to play a leading role in realizing optical to microwave quantum transducers for interconnecting future superconducting quantum machines via optical fibres^4^,^5^.

Scalable and robust QLMIs require emitters to have long spin coherence times and coherent optical transitions. For integrated optical quantum networks, these emitters need to be coupled to on-chip optical resonators that capture the photons in a single mode and further couple them into optical fibres or waveguides. The solid-state emitters most investigated so far for on-chip QLMIs are semiconductor quantum dots (QDs)^6^ and nitrogen vacancy (NV) centres in diamond^7^. To date, complete quantum control of single QD and NV spins, spin–photon entanglement and entanglement of remote NVs via photons have been realized^8^,^9^,^10^. Both QDs^11^ and NVs^12^ have been coupled to optical nanocavities. However, the challenge in growing optically identical QDs limits their prospects for a scalable architecture^6^. NVs embedded in nanostructures have long electronic spin coherence times^13^, but suffer from optical spectral instabilities such as blinking and spectral diffusion^14^. These spectral instabilities have so far impeded the coherent coupling between optical fields and NV centres in nanoresonators that are essential for further developments of QLMIs.

Rare-earth ions (REIs) embedded in host crystals at cryogenic temperatures exhibit highly coherent quantum states in the 4*f* orbital^15^. The Zeeman or hyperfine states of REIs can have coherence times as long as 6 hours^16^, the longest ever demonstrated in a solid. These states are connected via optical transitions with the narrowest linewidth in the solid state (sub-kHz) and small inhomogeneous broadening (MHz to GHz)^17^. This outstanding optical and spin coherence makes REI-doped crystals the state-of-the-art material for macroscopic solid-state optical quantum memories^18^,^19^. Integrated REI-doped waveguide quantum memories have also been developed^20^,^21^. Detection and control of single REI spins has been recently demonstrated in bulk material, but not using the transitions employed in optical quantum memories at cryogenic temperatures^22^,^23^. Coupling the highly coherent optical transitions of REIs to nanocavities will enable on-chip QLMIs, where REI ensembles act as quantum memories and single REIs act as qubits^24^.

Here we demonstrate high-cooperativity coupling of a neodymium (Nd^3+^) ensemble to photonic nanocavities fabricated directly in the yttrium orthosilicate (YSO) host crystal and show coherent optical control of REIs coupled to nanophotonic cavities. These results are enabled by the long coherence time and small inhomogeneous broadening of cavity-coupled REIs, which are essential properties that may lead to nanophotonic QLMIs with better prospects for scalability than those based on NVs and QDs.

## Results

### Photonic nanocavities in YSO

**Figure 1:**
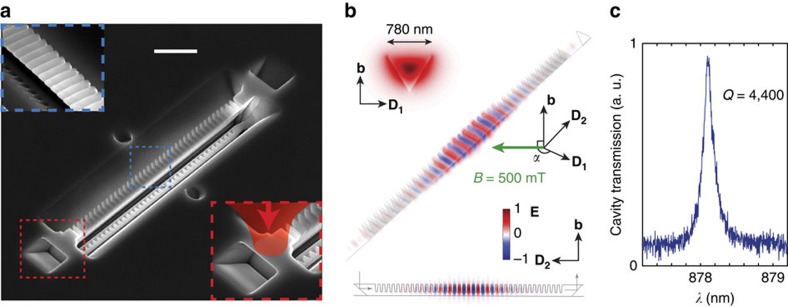
Photonic crystal nanobeam resonator fabricated in Nd:YSO. (**a**) Scanning electron microscope image of the device. Scale bar, 2 μm. The red inset is a zoomed-in view of the 45° angle-cut coupler that allows vertical coupling of light from a microscope objective. The blue inset shows the grooves forming the photonic crystal. (**b**) Schematics of the nanobeam resonator with simulated electric field (**E** along **D**_1_) profiles of the fundamental TE resonance mode. The TE polarization aligns with the **D**_1_ axis of the YSO crystal. A magnetic field of 500 mT is applied in the **D**_1_–**D**_2_ plane at an angle of *α*=135° with respect to the **D**_1_ axis. (**c**) Broadband cavity transmission spectrum showing the cavity resonance with quality factor *Q*=4,400.

The nanocavities, one of which is shown in Fig. 1a, were fabricated in neodymium-doped YSO (Nd^3+^:YSO) using focused ion beam milling. For this study, we used devices fabricated in two types of samples with Nd doping of 0.2% and 0.003% (Scientific Materials Inc.). The photonic crystal cavity is made of grooves milled in a triangular nanobeam^25^ (Fig. 1b) (see Methods). Finite-difference time-domain simulations^26^ indicate a transverse electric (TE) mode with quality factor exceeding 1 × 10^5^, mode volume *V*_mode_=1.65(*λ*/*n*_YSO_)^3^=0.2 *μ*m^3^ and mode profile shown in Fig. 1b. Here *V* is defined as *V*_mode_=∫_*V*_*ɛ*(**r**)|E(**r**)|^2^d^3^**r**/max(*ɛ*(**r**)|E(**r**)|^2^), where E(**r**) is the electric field and *ɛ*(**r**) is the electric permittivity at position **r**. Two 45° angled cuts at both ends of the nanobeam (that is, couplers) allow for coupling light from a direction normal to the chip (that is, b in Fig. 1b) using a confocal microscope setup (see Methods). A broadband light source was coupled into the resonator from one end and the transmitted light was collected from the other coupler with typical efficiencies ranging from 20% to 50%. The transmitted spectrum shows a resonance with quality factor *Q*=4,400 (Fig. 1c) in the device used for the following measurements. Arrays of devices were reproducibly fabricated with similar performance (Supplementary Fig. 1 and Supplementary Note 1).

### Coupling rate between REIs and the nanocavity

**Figure 2:**
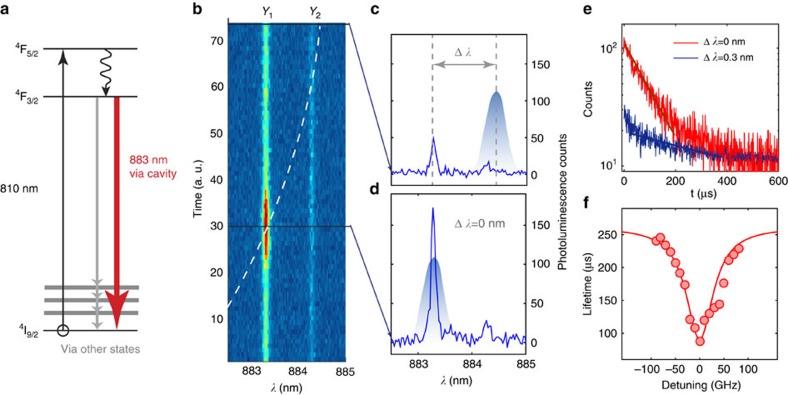
Purcell-enhanced coupling of Nd^3+^ ions to the YSO cavity mode. (**a**) Schematic of energy levels for Nd^3+^ in YSO. Optical excitation at 810 nm results in PL at several wavelengths with only the 883-nm transition enhanced by the cavity. (**b**) Spectrogram showing the Nd^3+^ PL, while the cavity is tuned across resonance using gas condensation. The dashed line is a guide to the eye indicating the central wavelength of the cavity resonance. The cavity resonance is not visible, because there is no background luminescence to populate the cavity mode. PL spectra in the uncoupled (**c**) and coupled (**d**) cases. The cavity resonance was drawn to indicate the cavity location. (**e**) Lifetime measurements for coupled (*τ*^*c*^=87 μs, Δ*λ*=0) and uncoupled (*τ*^0^=254 μs, Δ*λ*=0.3 nm) cases. (**f**) Change in lifetime as a function of the cavity detuning, which fits well with the calculation (red curve) using quality factor *Q*=4,400, 4.5% branching ratio and field intensity averaged over the mode volume.

The coupling of Nd^3+^ ions to the nanocavity was observed through enhancement in photoluminescence (PL) and emission rates. With the 0.2% device cooled at 3.5 K (Montana Instruments cryostation), an 810 nm laser coupled into the cavity excited PL in the ^4^I_9/2_–^4^F_3/2_ transition that was then collected from the output coupler (Fig. 2a). The PL spectrum shows two lines at 883.05 and 884.06 nm, corresponding to two inequivalent sites (*Y*_1_ and *Y*_2_) of Nd^3+^ in YSO. An important observation is that the inhomogeneous linewidth of the ions in the cavity is the same as in the bulk material, for both the 0.2% (Δ_inhom_=16.0 GHz) and 0.003% (5.9 GHz) devices (Supplementary Note 2). A small inhomogeneous linewidth (on the order of ∼10 GHz) is important for scaling to networks of multiple QLMIs (Supplementary Note 3). The cavity resonance was tuned across the Nd^3+^ PL line using a gas condensation technique^12^. The spectrograms in Fig. 2b–d show enhancement of the *Y*_1_ line when the cavity is resonant with it, which is a signature of coupling. The *Y*_2_ line exhibits negligible enhancement, because its dipole moment is not aligned with the TE cavity polarization (*D*_1_ axis of the YSO crystal, Fig. 1b). The spontaneous emission rate enhancement was characterized via lifetime measurements. A pulsed laser at 810 nm excited fluorescence of the *Y*_1_ line, which was filtered using a monochromator and detected with a single photon counter (Fig. 2e). From single exponential fits, we calculated a reduction in lifetime from 254 μ*s* when the cavity is detuned by Δ*λ*=0.3 nm, to 87 μ*s* on resonance. Taking into account the branching ratio of the 883 nm transition (*β*∼4.5%, see Methods), the reduction in lifetimes corresponds to an ensemble averaged Purcell factor^27^ *F*=42, which agrees well with the estimations that assume a uniform spatial distribution of Nd^3+^ ions in the resonator (Supplementary Note 4). A single ion positioned at the maximum cavity field would experience a Purcell factor of ∼200. A similar result was obtained in a 0.003% cavity with lower quality factor (Supplementary Fig. 2 and Supplementary Note 5).

### Optical coherence time for cavity-coupled REIs

**Figure 3:**
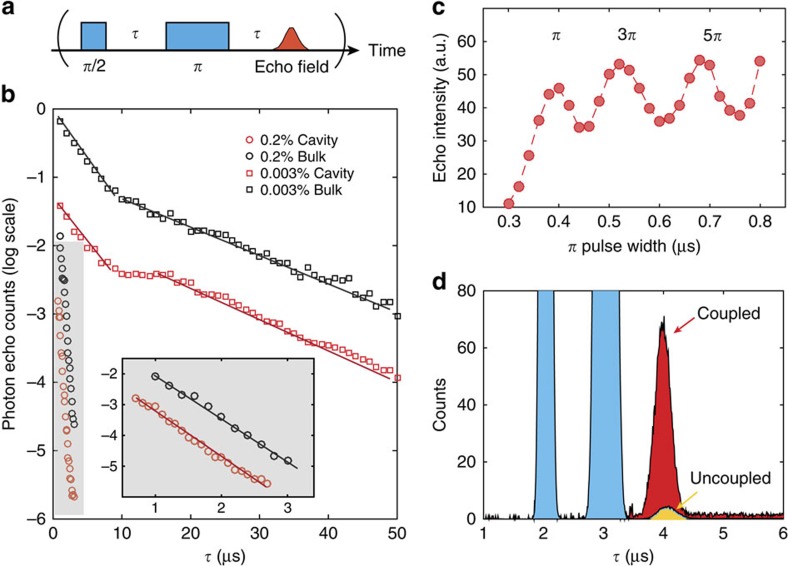
Photon echo measurements from an ensemble of Nd^3+^ ions in the cavity. (**a**) Two-pulse photon echo sequence (*π*/2−*π*) used to measure *T*_2_. (**b**) Two-pulse photon echo decays measured in both the cavity (red) and the bulk (black) samples with two different doping concentrations. The inset plots the echo decays measured with a 0.2% doped sample. (**c**) Oscillation of echo intensity with increasing width of the *π* rephasing pulse. The periodic signal reveals the ensemble averaged Rabi frequency of the coupled ions. The ideal *π* pulse duration for the input power was 0.4 μs. (**d**) Enhanced photon echo intensity (by ∼12-fold) when the cavity is coupled, compared with the uncoupled case (cavity detuned by Δ*λ*=15 nm so that the transition is outside the photonic bandgap).

Coherent and stable optical transitions are essential for QLMIs. We characterized the optical coherence time *T*_2_ of the 883-nm transition using two-pulse (*π*/2−*π*) photon echo techniques (Fig. 3a), with an applied magnetic field of **B**=0.5 T (see Methods). The laser pulses were coupled in and the echoes were collected via the couples when the 0.2% and 0.003% cavities were on resonance with the Nd transition. As only a small sub-ensemble (<100 ions) in the cavity was excited, the weak echo signal required detection using single photon counters. A typical echo from the 0.2% cavity is shown in Fig. 3d. The echo decays as a function of the (*π*/2−*π*) time delay *τ* are plotted in Fig. 3b together with photon echoes from bulk substrates. Echo intensity decays by e for delay τ_1/*e*_. For the 0.2% sample, 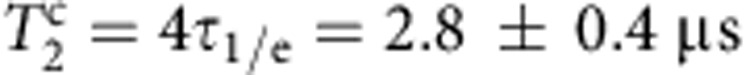 was measured for the cavity, which shows a good agreement with the bulk value of 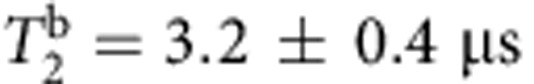. For the 0.003% sample, the echo exhibited two exponential decays. The slower decays give 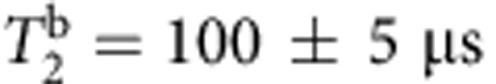 (bulk) and 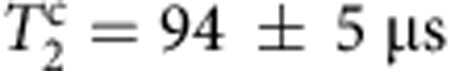 (cavity), which match with values reported in ref. 28. The fast decays are likely due to the superhyperfine interactions between Nd^3+^ and its neighbouring yttrium ions, which commonly manifests as modulated echoes decaying faster than *T*_2_ (ref. 28). No oscillations were observed in Fig. 3a because of the fast modulation frequency (∼1 MHz) due to the strong magnetic field. Oscillations of echoes for the initial 10 μs delay were observed when the **B** field was reduced to <100 mT. In addition, changes in the *T*_2_ values were not observed as the excitation power was varied, which indicates the measurement was not significantly affected by instantaneous spectral diffusion. In sum, the good agreement on *T*_2_ between the cavity and bulk confirms that the optical coherence property of Nd^3+^ ions is not affected by the nanofabrication. For higher Purcell factors, the *T*_2_ in cavities should decrease owing to the *T*_2_≤2*T*_1_ limit and would become smaller than the bulk value. This regime is not reached here, because the Purcell enhanced 2*T*_1_ is not smaller than 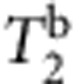.

The observation of photon echoes demonstrates coherent optical control of the quantum state of cavity-coupled ions. This control was further extended by varying the *π* pulse duration and observing Rabi oscillations in the echo intensities as shown in Fig. 3c. A Rabi frequency of ∼6 MHz is estimated. The same oscillation was not observed in the bulk. For the coupled laser power, the optimal *π* pulse duration is 0.4 μs. The oscillations are not visible for pulse duration <0.3 μs, because of the limited rise/fall times (∼200 ns) of the pulse-generating setup (Methods). A ∼12-fold increase in the echo intensity is observed in the cavity-coupled case compared with the uncoupled case (that is, detuning Δ*λ*=15 nm) as shown in Fig. 3d. This enhancement can be attributed to a combination of several effects: the higher atomic absorption rate through the Purcell effect^29^, stronger intracavity field intensity and high echo collection efficiency as the ions emit dominantly into the cavity mode. The spectral diffusion of the coupled ions using three-pulse photon echoes was also investigated. The homogeneous linewidths were broadened at rates of 6.1 kHz μs^−1^ for the 0.2% doped cavity and 380 Hz μs^−1^ for the 0.003% cavity. These slow spectral diffusions permit repeated optical addressing of the ions for 10 s of *μ*s (Supplementary Fig. 3 and Supplementary Note 6).

### Dipole-induced transparency and statistical fine structure

**Figure 4:**
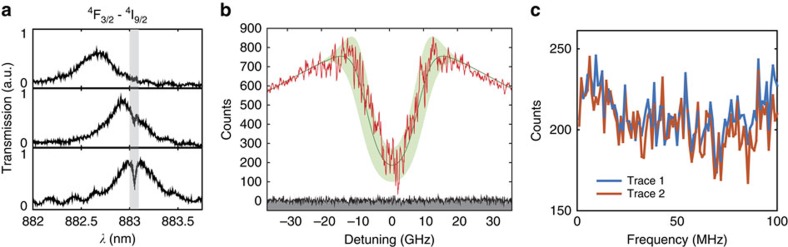
Control of cavity transmission and observation of SFS. (**a**) Broadband transmission spectra as the cavity is tuned to the 883 nm Nd transition. A dip is observed when the two are on resonance. The negligible dip at far detunings confirms that this effect is not due to absorption, but quantum interference between the intracavity field and the ions. (**b**) High-resolution transmission spectrum (red curve) obtained by scanning a narrow linewidth (∼20 kHz) Ti:Sapphire laser over the inhomogeneous line. The green curve is the fit using parameters: 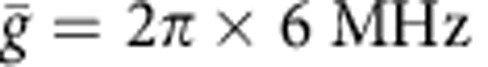, Γ_h_=2π × 100 kHz, Γ_inhom_=16.0 GHz, and assuming a Gaussian ion density distribution. The green shaded region is the estimated fluctuation in the transmitted laser intensity caused by 
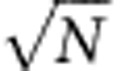
 statistical variations of the ion density. Large fluctuations are expected, because the density *N* is low (few tens), which agrees with the measurement. The fluctuation within the inhomogeneous linewidth is noticeably larger than that at far detunings (>25 GHz) and the technical background noise (grey area), confirming that they are caused by SFS of the ion spectral density. (**c**) Two traces of the transmitted intensities over the same 100 MHz bandwidth near zero detuning at different times. The high degree of correlation confirms the static and repeatable nature of SFS.

QLMIs require efficient interactions between atoms and photons, which is why quantum memories use long atomic clouds or doped crystals to achieve large optical depth. One key advantage provided by nanoresonators is that efficient atom–photon interaction can be achieved in a small volume with only a handful of ions. This is readily observable in our system, where the Nd^3+^ ions coherently interact with the intracavity field and control its transmission via dipole-induced transparency^30^. With the cavity tuned to 883 nm, the cavity transmission was probed using broadband light and a dip was observed at resonance (Fig. 4a). The depth of the dip depends on the collective coupling cooperativity 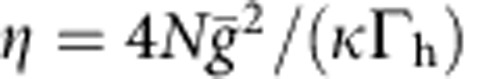, where 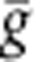 is the ensemble averaged coupling strength, *κ* is the cavity full linewidth, Γ_h_ is the Nd^3+^ homogeneous linewidth and *N* is the number of ions per Γ_h_. Considering an empirical collective dipole–cavity coupling model, the normalized cavity transmission in the presence of unsaturated resonant ions is,


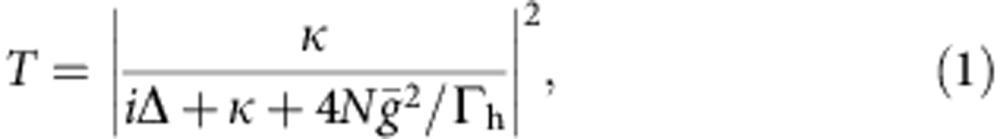


which simplifies to *T*=(1+*η*)^−2^ for zero detuning^31^. The cavity transmission can be controlled by varying the probe light power and observing the saturation of the ions at increasing intracavity photon number (Supplementary Fig. 4 and Supplementary Note 7). The saturation photon number in the nanocavity was measured to be 〈*n*_cav_〉=2 × 10^−5^.

To better resolve the spectrum, a narrow (∼20 kHz) Ti:Sapphire laser was scanned across the resonance (see Methods) to give the transmitted signals shown in Fig. 4b,c. A 75% decrease in transmission was measured at zero detuning, which corresponds to a collective cooperativity *η* ∼1.2. Fitting using a Gaussian spectral density distribution (green line) with measured parameters 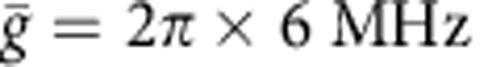, Γ_h_=2π × 100 kHz and Γ_inhom_=16.0 GHz gives a peak ion density of *N*≈53. Because of the statistical fine structure (SFS) of the inhomogeneously broadened line, a variation in the transmitted intensity (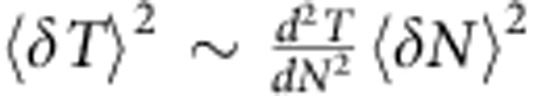), owing to 
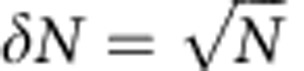
 fluctuations in the ion spectral density is expected^32^. This expected variation is represented in Fig. 4b by the green shaded region and shows good agreement with that of the measured signal. This variation within the inhomogeneously broadened line, on which the statistical fluctuations of the ion spectral density are imprinted, is significantly larger than the spectrometer technical noise (grey area) and laser shot noise at far detunings (>25 GHz), thus confirming that the static SFS in spectral density *N*(Δ*λ*) is probed. Two traces of the laser scan over the same 100 MHz bandwidth near zero detuning at different times are shown in Fig. 4c. The high degree of correlation reflects the static and repeatable nature of SFS. Notably, the current platform would allow detection and control of a single ion coupled to the cavity if *N*<<1 and the laser linewidth were narrower than Γ_*h*_ (Supplementary Fig. 5 and Supplementary Note 8).

## Discussion

The results reported in this paper (long optical coherence time, small inhomogeneous broadening, enhanced coherent optical control and resonant probing of cavity-coupled REIs) demonstrate REI-based nanophotonics as a promising approach for robust and scalable quantum photonic networks integrating memories and single REI qubits. Single photon rates exceeding 1 MHz can be achieved with single REIs in nanocavities with *Q*/*V*∼10^4^–10^5^ (*V* is normalized to (*λ*/*n*)^3^) and the inhomogeneous broadening allows for frequency multiplexing of multiple REIs. To use the interface as an optical quantum memory, efficient optical pumping into the long-lived Zeeman level needs to be demonstrated. Bulk REI quantum memories already boast high storage efficiency^33^ with multi-mode capacity^28^. Their implementations in our nanophotonic platform open the possibility of multiplexed systems for on-chip quantum repeaters. For Nd, high-fidelity storage of entanglement based on atomic frequency comb has been demonstrated^34^,^35^. With cavity impedance matching^29^, unit storage efficiency is achievable with a mesoscopic ensemble of cavity-coupled ions. Meanwhile, long-lived nuclear spin coherence of 9 ms in ^145^Nd (ref. 36) bodes well for spin–wave quantum memories using our nanoresonators. These devices can be further coupled to superconducting or optomechanical devices, to enable hybrid quantum systems^4^. Furthermore, the technology can be readily transferred to other wavelengths, such as 1.5 μm for telecom quantum memories using Er^3+^:YSO or 580 nm for long-haul quantum hard drives using Eu^3+^:YSO (ref. 16).

## Methods

### YSO nanoresonator design and fabrication

The nanobeam has an equilateral triangular cross-section with each side of 780 nm. This geometry allows a circular fundamental mode field that can be efficiently coupled with a free space laser beam. The cavity is formed by 40 equally spaced grooves of lattice constant 340 nm on the nanobeam, except for a defect introduced at the centre by perturbing the lattice constant. The depth of the grooves is 65% of the beam height. The triangular nanobeam resonator was fabricated using focused ion beam milling followed by wet etching of Ga^+^ contaminated YSO in diluted (10%) hydrochloric acid. An ion beam of 20 kV, 0.2 nA was used to fabricate the suspended nanobeam waveguide by milling at 30° angle with respect to the crystal surface normal. A small ion beam of 23 pA was then used to accurately pattern the grooves on top of the nanobeam. Limited by the finite width of the focused ion beam, the side walls of the grooves in the actual device were not vertical, but had an angle of 6°. This leads to a degraded theoretical *Q* of 5.0 × 10^4^. We were able to reproducibly fabricate arrays of resonators (up to six) in a batch (Supplementary Fig. 1), with all the devices measuring resonances close to 883 nm and quality factors varying from 1,100 to 10,000.

### Experimental setup for the photon echo measurements

A 500-mT external magnetic field was applied at *α*=135° relative to the crystal *D*_1_ axis using a pair of permanent magnets (see Fig. 1b). The *π*/2 and *π* Gaussian pulses were generated by amplitude-modulating the Ti:Sapphire laser with two acousto-optic modulators (AOMs) in series, with each in a double-pass configuration. The two pulse widths were 250 and 400 ns at a repetition rate of 1 kHz. The average (peak) power of the excitation pulses was 210 nW (320 μW) measured after the objective lens. The extinction ratio between the on and off level of the pulses was ∼120 dB, ensuring sufficient signal-to-noise ratio for detecting echo photons using a Si single-photon counter (Perkin Elmer SPCM). A third shutter AOM in single-pass configuration was inserted just before the photon counter to block the strong excitation pulses from saturating the detector. The extinction ratio of this shutter AOM was 30 dB.

### High-resolution laser spectroscopy on cavity-coupled Nd^3+^ ions

For the cavity transmission experiments, the Ti:Sapphire laser (M Squared SolsTiS) was continuously scanned at a rate of 10 MHz per second. The high-sensitivity charge-coupled device camera in the spectrometer (Princeton Instruments PIXIS) registers the transmitted photon counts intermittently at an adjustable frame rate (frames per second (fps)) with an exposure time 0.01 s for each frame. Therefore, one exposure corresponds to a spectral width of 10 MHz × 0.01=100 kHz scanned by the laser, which is equal to the homogeneous linewidth of the 0.2% doped sample. The long-term drift of the laser is 10 MHz per hour, so the drift during each exposure should be inconsequential. Each data point in Fig. 4b,c represents the photon count collected in one camera exposure, corresponding to the signal contributed by the ions within one homogeneous linewidth. The frame rate was 0.1 fps for the coarser scan in Fig. 4b, corresponding to a spectral interval of 100 MHz between two adjacent data points. The frame rate was 8.2 fps for the fine scan in Fig. 4c, with a spectral interval ∼1 MHz. Each data point was obtained with one scan. Several scans at different spectral regions were performed and stitched together to cover the entire bandwidth in Fig. 4b.

### Estimation of the branching ratio

The measured optical depth of a 15-*μ*m-long nanobeam resonator at 3.8 K was *d*=0.1, from which we deduce an oscillator strength of *f* = 6.5 × 10^−7^ and a spontaneous emission rate of this transition to be *γ*_883_=1/*τ*_883_=1/5.6 ms (ref. 24). With a measured bulk medium lifetime *τ*_0_=250 μs, the branching ratio was thus estimated to be *τ*_0_/*τ*_883_≈4.5%.

## Additional information

**How to cite this article:** Zhong, T. *et al*. Nanophotonic coherent light–matter interfaces based on rare-earth-doped crystals. *Nat. Commun.* 6:8206 doi: 10.1038/ncomms9206 (2015).

## Supplementary Material

Supplementary Information Supplementary Figures 1-5, Supplementary Notes 1-8 and Supplementary References

## References

[b1] Kimble H. J. The quantum internet. Nature 453, 1023–1030 (2008). 18563153 10.1038/nature07127

[b2] Kómár P. . A quantum network of clocks. Nat. Phys. 10, 582–587 (2014).

[b3] Chen W. . All-optical switch and transistor gated by one stored photon. Science 314, 768–770 (2013). 23828886 10.1126/science.1238169

[b4] Williamson L. A., Chen Y.-H. & Longdell J. J. Magneto-optic modulator with unit quantum efficiency. Phys. Rev. Lett. 113, 203601 (2014). 25432041 10.1103/PhysRevLett.113.203601

[b5] Andrews R. W. . Bidirectional and efficient conversion between microwave and optical light. Nat. Phys. 10, 321–326 (2014).

[b6] Michler P. (ed.) Single Quantum Dots Springer Berlin Heidelberg (2009).

[b7] Aharonovich I. & Neu E. Diamond nanophotonics. Adv. Optical Mater. 2, 911–928 (2014).

[b8] Bernien H. . Heralded entanglement between solid-state qubits separated by three metres. Nature 497, 86–90 (2013). 23615617 10.1038/nature12016

[b9] Press D., Ladd T. D., Zhang B. & Yamamoto Y. Complete quantum control of a single quantum dot spin using ultrafast optical pulses. Nature 456, 218–221 (2008). 19005550 10.1038/nature07530

[b10] De Greve K. . Quantum-dot spin-photon entanglement via frequency downconversion to telecom wavelength. Nature 491, 421–425 (2012). 23151585 10.1038/nature11577

[b11] Englund D. . Controlling cavity reflectivity with a single quantum dot. Nature 450, 857–861 (2007). 18064008 10.1038/nature06234

[b12] Faraon A., Barclay P. E., Santori C., Fu K. C. & Beausoleil R. G. Resonant enhancement of the zero-phonon emission from a colour centre in a diamond cavity. Nat. Photon. 5, 301–305 (2011).

[b13] Li L. . Coherent spin control of a nanocavity-enhanced qubit in diamond. Nat. Commun. 6, 6173 (2015). 25629223 10.1038/ncomms7173

[b14] Faraon A., Santori C., Huang Z., Acosta V. M. & Beausoleil R. G. Coupling of nitrogen-vacancy centers to photonic crystal cavities in monocrystalline diamond. Phys. Rev. Lett. 109, 033604 (2012). 22861849 10.1103/PhysRevLett.109.033604

[b15] Thiel C., Böttger T. & Cone R. Rare-earth-doped materials for applications in quantum information storage and signal processing. J. Luminesc. 131, 353–361 (2001).

[b16] Zhong M. . Optically addressable nuclear spins in a solid with a six-hour coherence time. Nature 517, 177–180 (2015). 25567283 10.1038/nature14025

[b17] Sun Y., Thiel C. W., Cone R. L., Equall R. W. & Hutcheson R. L. Recent progress in developing new rare earth materials for hole burning and coherent transient applications. J. Lumin. 98, 281–287 (2002).

[b18] Lvovsky A. I., Sanders B. C. & Tittel W. Optical quantum memory. Nat. Photon. 3, 706–714 (2009).

[b19] Tittel W. . Photon-echo quantum memory in solid state systems. Laser Photon. Rev. 4, 244–267 (2010).

[b20] Saglamyurek E. . Broadband waveguide quantum memory for entangled photons. Nature 469, 512–515 (2011). 21228775 10.1038/nature09719

[b21] Marzban S., Bartholomew J. G., Madden S., Vu K. & Sellars M. J. Observation of photon echoes from evanescently coupled rare-earth ions in a planar waveguide. Phys. Rev. Lett. 115, 013601 (2015). 26182097 10.1103/PhysRevLett.115.013601

[b22] Kolesov R. . Optical detection of a single rare-earth ion in a crystal. Nat. Commun. 3, 1029 (2012). 22929786 10.1038/ncomms2034 PMC3432461

[b23] Utikal T. . Spectroscopic detection and state preparation of a single praseodymium ion in a crystal. Nat. Commun. 5, 3627 (2014). 24722142 10.1038/ncomms4627

[b24] McAuslan D. L. & Longdell J. J. Cavity QED using rare-earth-metal-ion dopants in monolithic resonators: what you can do with a weak oscillator. Phys. Rev. A 80, 062307 (2009).

[b25] Bayn I., Meyler B., Salzman J. & Kalish R. Triangular nanobeam photonic cavities in single-crystal diamond. N. J. Phys. 13, 025018 (2011).

[b26] Oskooi A. F. . MEEP: a flexible free-software package for electromagnetic simulations by the FDTD method. Comput. Phys. Commun. 181, 687–702 (2010).

[b27] Purcell E. M. Spontaneous emission probabilities at radio frequencies. Phys. Rev. 69, 681 (1946).

[b28] Usmani I., Afzelius M., de Riedmatten H. & Gisin N. Mapping multiple photonic qubits into and out of one solid-state atomic ensemble. Nat. Commun. 1, 12 (2010). 20975673 10.1038/ncomms1010

[b29] Afzelius M. & Simon C. Impedance-matched cavity quantum memory. Phys. Rev. A 82, 022310 (2010).

[b30] Waks E. & Vucković J. Dipole induced transparency in drop-filter cavity-waveguide systems. Phys. Rev. Lett. 96, 153601 (2006). 16712156 10.1103/PhysRevLett.96.153601

[b31] Thompson J. D. . Coupling a single trapped atom to a nanoscale optical cavity. Science 340, 1202–1205 (2013). 23618764 10.1126/science.1237125

[b32] Moerner W. E. & Carter T. P. Statistical fine structure of inhomogeneously broadened absorption lines. Phys. Rev. Lett. 59, 2705 (1987). 10035627 10.1103/PhysRevLett.59.2705

[b33] Hedges M. P., Longdell J. J., Li Y. & Sellars M. J. Efficient quantum memory for light. Nature 465, 1052–1056 (2010). 20577210 10.1038/nature09081

[b34] Clausen C. . Quantum storage of photonic entanglement in a crystal. Nature 469, 508–511 (2011). 21228774 10.1038/nature09662

[b35] Bussières F. . Quantum teleportation from a telecom-wavelength photon to a solid-state quantum memory. Nat. Photon. 8, 775–778 (2014).

[b36] Wolfowicz G. . Coherent storage of microwave excitations in rare earth nuclear spins. Phys. Rev. Lett. 114, 170503 (2015). 25978214 10.1103/PhysRevLett.114.170503

