# Supplementary material for: Nanophotonic coherent light–matter interfaces based on rare-earth-doped crystals
Source: Nat Commun. 2015 Sep 14;6:8206. doi: 10.1038/ncomms9206 (PMC4647856; doi:10.1038/ncomms9206)
Supplement: Supplementary Information — Supplementary Figures 1-5, Supplementary Notes 1-8 and Supplementary References [file ncomms9206-s1.pdf]

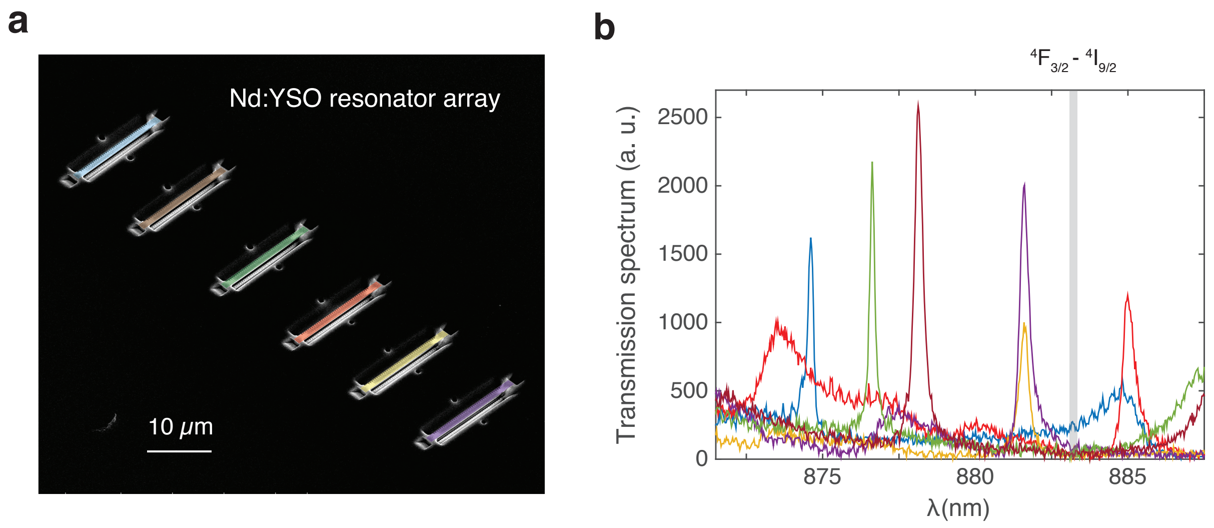

Supplementary Figure 1. An array of nano-beam resonators fabricated in Nd:YSO. (**a**) Scanning electron microscope image of 6 devices. (**b**) Measured resonance modes (color matched to the corresponding device) near the 883 nm transition of Nd:YSO (grey line).

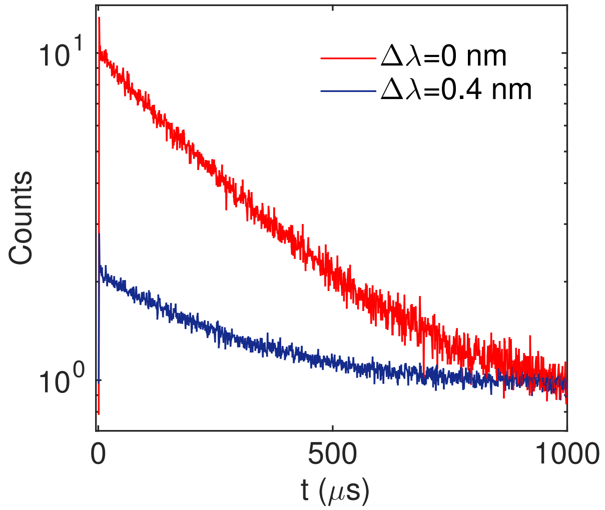

Supplementary Figure 2. Lifetime measurements for a 0.003% doped Nd:YSO resonator. Lifetime changes from 289 μs when the cavity of Q=1,500 is detuned by Δ=0.4 nm, to 200 μs when the cavity is resonant with the ions.

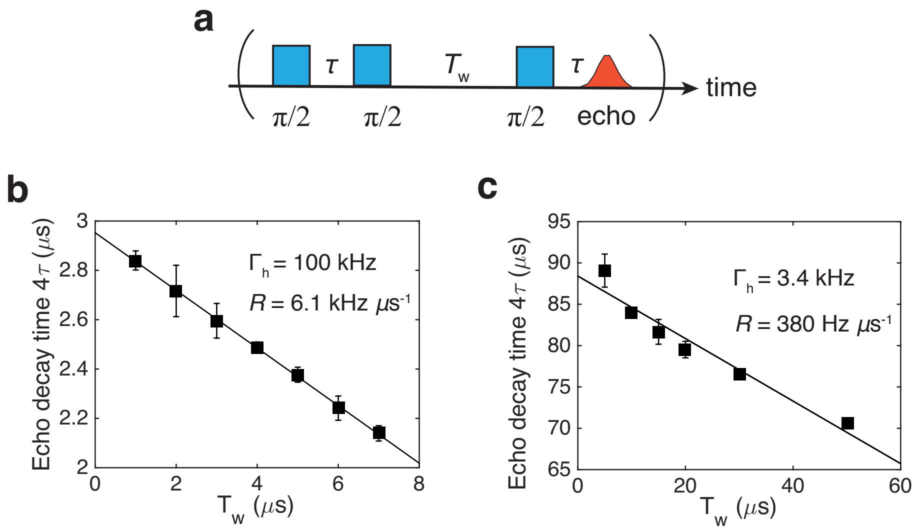

Supplementary Figure 3. **Spectral diffusion of cavity-coupled ions** (**a**) Three-pulse photon echo sequence ($\pi/2$ - $\pi/2$ - $\pi/2$). (**b, c**) T_{w} dependent broadening of the effective linewidths of ions coupled to the 0.2% (**b**) and 0.003% (**c**) doped cavities, measured via three-pulse photon echoes. Linear fits indicate the spectral diffusion rates R for both doping concentrations.

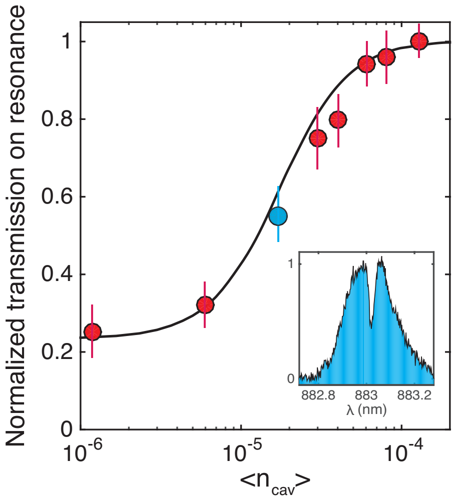

Supplementary Figure 4. **Controlled cavity transmission versus intra-cavity photon number for probe laser at zero detuning.** The cavity transmission is normalized by empty-cavity transmission at the zero detuning. Saturation occurs at $\langle n_{\mathrm{cav}} \rangle \approx 2 \times 10^{-5}$. The inset shows a transmission spectrum at onset of saturation with a normalized transmission at the dip of ≈54%.

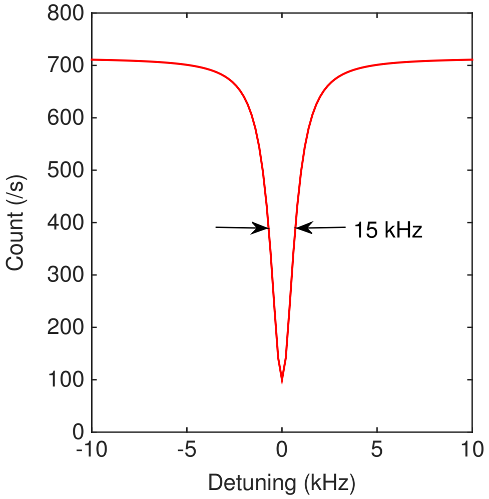

Supplementary Figure 5. **Cavity transmission when coupled to a single Nd^{+3} ion.** The simulation uses Quantum Optics Toolbox [5] with parameters Q=4,400, Γ_h=3.1 kHz, and g= $2\pi\times10$ MHz. The transmission dip has a full-width at half-maximum (FWHM) of $\approx$15 kHz.

Supplementary Note 1.

Fabrication and characterization of arrays of YSO nano-beam resonators. The YSO nano-resonators were fabricated in batch with careful focused ion beam (FIB) alignment and drift compensation. Supplementary Figure 1a shows a SEM image of an array of six Nd:YSO nano-resonators. All of the devices have resonance modes near the designed 883 nm wavelength, shown in the transmission spectrum in Supplementary Figure 1b. The color of each spectrum maps to that of the device in Supplementary Figure 1a. Measured quality factors in this batch range from 1,500 to 4,400. The spread of resonance wavelengths is about 10 nm, indicating the robustness of this fabrication process.

Supplementary Note 2.

Measurements of inhomogeneous linewidths for cavity-coupled ions. The inhomogeneous linewidth for the ions coupled to the 0.2% nano-cavity was measured to be Δ_{inhom}=16.0 GHz from the dipole-induced transparency signal in Fig. 4b. For the 0.003% cavity, the ion density was too low for a similar measurement of the inhomogeneously broadened distribution. Instead, the linewidth of photoluminescence (PL) from the cavity was

measured with a high resolution spectrometer, and Δ_{inhom}=5.9 GHz was estimated by deconvolving the PL signal with the minimally resolvable linewidth of the spectrometer. For both doping levels, the same inhomogeneous linewidths were measured from the bulk via absorption spectroscopy. The agreement between the inhomogeneous linewidth of the cavity-coupled Nd ions and the bulk confirms the excellent spectral stability of REIs when embedded in nanophotonic resonators.

Supplementary Note 3.

Requirement on the inhomogeneous linewidths for scalable QLMIs. We consider a network of QLMIs each being a nano-cavity coupled to ensembles of emitters with inhomogeneous linewidth Δ_{inhom}. Efficient QLMIs require the emitters to emit photons dominantly into the cavity mode. The cavity photons in a single spatial mode could then be efficiently coupled to waveguides or fibres for routing to other QLMIs operating at the same frequency. The probability of an emitter to emit a photon into the cavity mode is $\beta F/(1+(F-1)\beta)$, where β is the branching ratio of the dipole transition, and F is the Purcell factor in Eq. 2. Assuming 99% of the dipole emission into the cavity and typical branching ratio of $\beta \approx 10\%$, the required Purcell factor should be ∼1,000. Considering a photonic crystal nano-cavity with a small mode volume of $1(\lambda/n)^3$, $F \sim 1,000$ corresponds to a quality factor $Q \approx 1 \times 10^4$ and a cavity linewidth κ ≈30 GHz (for the 883 nm transition). Thus, for scalability, the emitters and cavities need to be aligned within ≈10 GHz (order of magnitude). This limits the inhomogeneous broadening of the dipole ensembles to be <10 GHz for implementing robust and scalable QLMIs. This condition is satisfied by most REI transitions.

Supplementary Note 4.

Calculation of ensemble averaged Purcell enhancement factor. The spontaneous emission rate of a dipole coupled to a nano-resonator is enhanced relative to the bulk medium, by the factor $\sim 1+\beta F$ [1], where β is the branching ratio of the transition, and F is given by [2],

$$F = F_{\text{cav}} \left(\frac{\mathbf{E}(\mathbf{r}) \cdot \mu}{|\mathbf{E}_{\text{max}}||\mu|} \right)^2 \frac{1}{1+4Q^2(\lambda/\lambda_{\text{cav}}-1)^2} \tag{1}$$

where μ is the dipole moment, $\mathbf{E}(\mathbf{r})$ is the local electric field at the emitter location r, $\lambda_{\rm cav}$ is the cavity resonant wavelength, λ is the emitter wavelength, and $|\mathbf{E}_{\rm max}|$ is the maximum electric field in the resonator. For a dipole that is resonant with the cavity and ideally positioned and oriented with respect to the maximum cavity field,

$$F_{\rm cav} = \frac{3}{4\pi^2}\left(\frac{\lambda_{\rm cav}}{n}\right)^3 \frac{Q}{V_{\rm mode}}. \tag{2}$$

We consider an ensemble of Nd ions uniformly distributed inside the YSO cavity. The enhancement of the emission from the ensemble can be estimated by averaging $F_{\rm cav}$ (Eq. 1) over the entire population of Nd ions in the cavity. Based on the 3 dimensional field profile in Fig. 1b, the mode volume $1.65(\lambda/n)^3$ and Q=4,400, we numerically calculate this averaged Purcell factor to be 45 when the cavity is resonant with the transition.

If the emission rate for uncoupled Nd ions is $1/\tau_0 = 1/\tau_{883} + 1/\tau_{\rm other}$, in the coupled case the rate becomes $1/\tau_{\rm c} = (1+F)/\tau_{883}+1/\tau_{\rm other}$, where $1/\tau_{883}$ and $1/\tau_{\rm other}$ are the spontaneous emission rates into the 883 nm transition and other 4f-4f transitions, respectively. The Purcell factor is then experimentally extracted as $F = (\tau_0/\tau_{\rm c}-1)/\beta$, where β is the branching ratio of the 883 nm line. Based on the measured branching ratio β= 4.5%, the observed change in lifetimes leads to an ensemble averaged Purcell factor $\sim$ 42, which matches well with the calculated value. Furthermore, the averaged value of 42 means the expected Purcell enhancement for an ideally positioned and oriented Nd dipole is $F_{\rm cav} \approx 200$.

Supplementary Note 5.

Purcell enhancement in 0.003% doped Nd:YSO nano-resonators. A 0.003% doped Nd:YSO nano-resonator was fabricated, measuring a resonance mode at 879 nm with quality factor Q=1,500. Spontaneous emission rate enhancement in this cavity was estimated from lifetime measurements in the same way as for the 0.2% cavity. As shown in Supplementary Figure 2, a change of lifetime from 289 μs when the cavity is detuned by Δ =0.4 nm, to 195 μs at resonance gives rise to an ensemble averaged Purcell factor $F \sim 10$. Note that a longer T_1 =290 μs in the low density Nd:YSO bulk sample (T_1 = 300 μs reported in [3]) yields a slightly larger branching ratio $\beta \sim 5.4\%$. The longer T_1 is most likely due to weaker dipole-dipole interactions in 0.003% doped sample.

Supplementary Note 6.

Spectral diffusion of Nd^{3+} ions coupled to the nano-cavities. The dynamic coherence properties of the cavity-coupled Nd ions were investigated by three-pulse photon echoes (($\pi/2$ - $\pi/2$ - $\pi/2$)) that gives information about the spectral diffusion on time scales up to T_1 [4] (Supplementary Figure 3a). The third pulse, delayed by a time T_{w} after second pulse, is diffracted on the spectral grating from the first two pulses and produces an echo. Spectral diffusion - frequency shifts of the optical transition due to the fluctuating rare earth environment - gradually erases the grating during T_{w}, and causes faster echo decays thus broadening of the effective linewidth Γ_{eff}. Linearly increasing $\Gamma_{\mathrm{eff}} = \Gamma_{\mathrm{h}} + RT_{\mathrm{w}}$ at a rate of $R = 380$ Hz $\mu\mathrm{s}^{-1}$ was measured for the 0.003% cavity (Supplementary Figure 3b) and 6.1 kHz $\mu\mathrm{s}^{-1}$ for the 0.2% cavity (Supplementary Figure 3c). Higher spectral diffusion is expected for higher doping because of stronger dipole-dipole interaction between Nd ions. Nevertheless, the measured linewidth broadening is much smaller than our Rabi frequency (∼6 MHz in Fig. 3c). This indicates the coupled ions, either singles or ensembles, can be optical addressed repeatedly up to 10s of μs, which is desirable for optical quantum information processing.

Supplementary Note 7.

REI-controlled cavity transmission and saturation of the coupled ions. Supplementary Figure 4 plots the on resonance transmission at zero detuning as a function of the average photon number in the cavity $\langle n_{\mathrm{cav}}\rangle$. $\langle n_{\mathrm{cav}}\rangle$ was estimated from the input probe laser power P_{in} (measured after the objective), the coupler efficiency η, and cavity coupling rate ($\kappa/2$) as $\langle n_{\mathrm{cav}}\rangle = \eta P_{\mathrm{in}}/\kappa\hbar\omega$. Black curve is the theoretical calculation using the Quantum toolbox [5], which shows close agreement with the experiment.

Supplementary Note 8.

Towards detection and control of single REI ions coupled to the nano-cavity. The measurement of $N(\Delta\lambda)$ indicate that this system can be used to detect and control a single ion coupled to the cavity - a key ingredient for realizing quantum networks interconnecting

multiple quantum bits encoded in individual REI ions. In the 0.003% low density devices, we estimated a peak ion density of N=0.07 per $\Gamma_{\mathrm{h}}=1/\pi T_2$=3.1 kHz. Correspondingly, the single ion cooperativity of η =1.6 can be attained with the same cavity Q=4,400, $V = 1.65(\lambda/n)^3$, and $g = 2\pi\times10$ MHz (typical for REI transitions and we assume the ion is positioned at maximum cavity field). Simulation using Quantum Optics Toolbox [5] yields a transmission dip >80% due to a single Nd ion, as shown in Supplementary Figure 5. The main technical challenge to detect single ion in this cavity system is the requirement of a highly stabilized laser, with linewidth <1 kHz and minimal long term drift, for scanning the single ion spectrum, which should be attainable with state of the art laser spectroscopy technology.

Supplementary References

[1] Faraon, A., Barclay, P. E., Santori, C., Fu, K. C., & Beausoleil, R. G. Resonant enhancement of the zero-phonon emission from a colour centre in a diamond cavity. *Nat. Photon.* **5,** 301-305 (2011).

[2] Purcell, E. M. Spontaneous emission probabilities at radio frequencies. *Phys. Rev.* **69,** 681 (1946).

[3] Usmani, I, Afzelius, M., de Riedmatten, H., & Gisin, N. Mapping multiple photonic qubits into and out of one solid-state atomic ensemble. *Nat. Commun.* **1,** 12 (2010).

[4] Perrot, A. *et al.* Narrow Optical Homogeneous Linewidths in Rare Earth Doped Nanocrystals. *Phys. Rev. Lett.* **111,** 203601 (2013).

[5] Tan S. M. A computational toolbox for quantum and atomic optics. *J. Opt. B: Quantum Semiclass. Opt.* **1,** 424 (1999).
